# *Dysosmavillosa* (Berberidaceae), a new species from Guizhou, Southwestern China

**DOI:** 10.3897/phytokeys.124.34435

**Published:** 2019-06-21

**Authors:** Zhiwei Wang, Wenguang Sun, Houcheng Xi, Shuai Chang

**Affiliations:** 1 College of Pharmacy, Guizhou University of Traditional Chinese Medicine, Guiyang 550002, Guizhou, China Department of Pharmacy, Guizhou University of Traditional Chinese Medicine Guiyang China; 2 Key Laboratory for Plant Diversity and Biogeography of East Asia, Kunming Institute of Botany, Chinese Academy of Sciences, Kunming 650201, Yunnan, China Key Laboratory for Plant Diversity and Biogeography of East Asia, Kunming Institute of Botany, Chinese Academy of Sciences Kunming China; 3 Xishuangbanna Tropical Botanical Garden, Chinese Academy of Sciences, Xishuangbanna 666303, Yunnan, China Xishuangbanna Tropical Botanical Garden, Chinese Academy of Sciences Xishuangbanna China; 4 Yunnan Key Laboratory for Integrative Conservation of Plant Species with Extremely Small Populations, Kunming Institute of Botany, Chinese Academy of Sciences, Kunming 650201, Yunnan, China unnan Key Laboratory for Integrative Conservation of Plant Species with Extremely Small Populations, Kunming Institute of Botany, Chinese Academy of Sciences Kunming China

**Keywords:** Asia, Berberidaceae, *
Dysosma
*, Podophylloideae, *
Podophyllum
*, Ranunculales

## Abstract

A new species, *Dysosmavillosa* Z.W.Wang & H.C.Xi, is described and illustrated based on collections from the Yueliang Mountains in Congjiang County of Guizhou, Southwestern China. It is morphologically similar to *D.difformis* (Hemsl. & E.H.Wilson) T.H.Wang *ex* T.S.Ying, but can be easily distinguished from *D.difformis* by its inflorescences bearing a greater number of flowers (5–13 *vs.* 2–5), white-villous stems, petioles, and abaxial leaf blade, and stigma dark purple-red. In addition, we also compare this new species to the three species of *Podophyllum* (i.e., *P.glaucescens* J.M.H.Shaw, *P.hemsleyi* J.M.H.Shaw & Stearn, and *P.trilobulum* J.M.H.Shaw) which are insufficiently known and listed as putative members of *Dysosma* in *Flora of China. Dysosmavillosa* can also be easily distinguished from *P.glaucescens* (7-flowered; to 40 cm tall) and *P.hemsleyi* (4-flowered; to 40 cm tall) by its inflorescences bearing a greater number of flowers, relatively smaller stature (9–23 cm tall), stems, petioles and abaxial leaf blade densely white-villose. Although the stems and petioles of *P.trilobulum* also possess fine short hairs, it can be easily distinguished from *D.villosa* by its trilobulate leaves, inflorescence with fewer flowers (2–5), and the position of inflorescence (inserted at or above midpoint on petiole of upper leaf).

## Introduction

*Dysosma* Woodson, a small genus of Berberidaceae, has long been used in traditional herbal medicine in East Asia due to the presence of podophyllotoxin, which has important biological activities, such as treating external genital warts (Beutner and Von 1990, Wang 1991, Petersen et al. 1995, Ying et al. 2011, Mao et al. 2014). It occurs at the Subtropical Evergreen Broadleaved Forest belt of China, being morphologically close to *Sinopodophyllum* (Royle) T.S.Ying, *Podophyllum* L., and *Diphylleia* Michx. Nonetheless, it is differentiated by comprising perennial herbs with creeping rhizomes, numerous fibrous roots, 3–9-parted or lobed-peltate leaves, umbellate inflorescences, and berries with numerous seeds (Stähelin and Von 1991, Ying et al. 2011, Mao et al. 2014). Recently, a large number of phylogenetic analyses revealed the monophyly of *Dysosma* and its sister relationship to the *Sinopodophyllum* and *Podophyllum* (Loconte and Estes 1989, Nickol 1995, Kim and Jansen 1998, Liu et al. 2002, Wang et al. 2007, Mao et al. 2014). In the "Flora of China" (FOC) treatment, seven species are recognised: *D.delavayi* (Franch.) Hu, *D.pleiantha* (Hance) Woodson, *D.tsayuensis* T.S.Ying, *D.aurantiocaulis* (Hand.-Mazz.) Hu, *D.majoensis* (Gagnep.) M.Hiroe, *D.versipellis* (Hance) M.Cheng *ex* T.S.Ying and *D.difformis* (Hemsl. & E.H.Wilson) T.H.Wang *ex* T.S.Ying (Ying et al. 2011). In addition, due to inadequate material, *FOC* also treats three insufficiently known species described under *Podophyllum* (i.e., *P.glaucescens* J.M.H.Shaw, *P.hemsleyi* J.M.H.Shaw & Stearn, and *P.trilobulum* J.M.H.Shaw), but that probably belong to *Dysosma* (Ying et al. 2011).

During a field trip to the Yueliang Mountains, Congjiang County, Guizhou Province, Southwestern China, in May 2015, an unknown species with densely white-villous stems, petioles and abaxial leaf blade, and dark purple-red stigma was found. After a detailed examination of the characters of our material and possible closely similar species in *Dysosma* (including the three species of *Podophyllum* which are putative members of *Dysosma*), we concluded that these specimens actually represent an undescribed species. Thus, due to its uniqueness in characters, it is formally described by us, below.

## Materials and methods

Field investigations were conducted in the locality of the type specimens and other adjacent areas of Guizhou. The morphological description of the new species was based on an examination of the dried specimens in herbaria and living plants in the field. The comparison with morphologically similar species was based on an extensive check of specimens deposited in A, E, GH, PE, KUN, IBSC, HIB, HGAS, IBK and NAS, as well as the protologues and descriptions in related literature (Woodson 1928, Kim and Jansen 1996, 1998, Wang et al. 2007, Ying et al. 2011).

## Taxonomic treatment

### Dysosma villosa

Taxon classificationPlantaeRanunculalesBerberidaceae

Z.W.Wang & H.C.Xi sp. nov.

urn:lsid:ipni.org:names:77198709-1

Figures 1 , 2 , 3

#### Diagnosis.

*Dysosmavillosa* is most similar to *D.difformis* (Hemsl. & E.H.Wilson) T.H.Wang *ex* T.S.Ying, but differs from the latter by its inflorescences generally with more flowers (5–13 vs. 2–5), white-villous petioles, stems and abaxial leaf blade and dark purple-red stigma.

#### Type.

CHINA. Guizhou: Congjiang County, Guanghui Town, Jiaya Village, Yueliang Mountains, alt. 1105 m, 25.636N, 108.293E, 09 May 2015, *Z.W. Wang & H.C. Xi WAZW15016* (holotype: CSH barcode CSH0160399!; isotype: KUN!).

#### Description.

Herbs 9–23 cm tall. Rhizomes usually terete, slender, with numerous fibrous roots. Stems erect, branched, pale green, white-villous. Leaves alternate, obliquely peltate; petioles 6–12 cm long, white-villous; blades 9–17 × 13–23 cm, papery, abaxially pale green, densely white-villous, adaxially deep green, glabrous, base not deeply divided or undivided, margin sparsely denticulate. Umbels 5–13-flowered, sessile, emerging near the base of the leaf blade. Flowers pendulous, pedicels 1.6–2.2 cm long, apically gibbous, sparsely white-villous; sepals 1.4–2.2 cm × 1–4 mm, oblong-lanceolate, pale green, abaxially pubescent, adaxially glabrous, apex acuminate; petals 4–5 × 1.2–1.6 cm, oblong-loriform, dark purple-red, glabrous, apex round; stamens 6, filaments flat, ca. 0.9 cm long, dark purple-red, anthers ca. 1.4 cm long, falcate, connectives exceeding the anther sacs in measurements, anther sacs, ca. 0.9 cm, dark purple, pollen yellow; ovary obpyriform, green, ca. 0.9 cm, style ca. 2 mm long, green, densely speckled with red or purple, stigma multilobate (crown-shaped), dark purple-red. Berry globose, 1.5–2.4 cm long. Seeds numerous, lacking an aril.

#### Distribution and habitat.

This new species is currently known from Yueliang mountains of Congjiang County, Guizhou Province, Southwestern China. It grows under forests, at an elevation between 800 and 1500 m.

#### Phenology.

This new species has been observed flowering from April to June and fruiting from June to September.

#### Etymology.

The specific epithet is derived from the character (white-villous petioles, stems and abaxial leaf blade) of this species.

#### Additional specimen examined.

**CHINA. Guizhou**: Congjiang County, Guanghui Town, Baiji Village, alt. 823 m, 25.633N, 108.291E, 20 April 2016, *Z.W. Wang & H.C. Xi WAZW16029* (KUN); Congjiang County, Guanghui Town, Changniu Village, alt. 936 m, 25.596N, 108.271E, 20 April 2016, *Z.W. Wang & H.C. Xi WAZW16032* (CSH); Ronjiang County, Jihua Town, Baiwang Village, alt. 806 m, 25.657N, 108.269E, 18 May 2017, *Z.W. Wang & H.C. Xi WAZW17024* (KUN).

**Figure 1.**
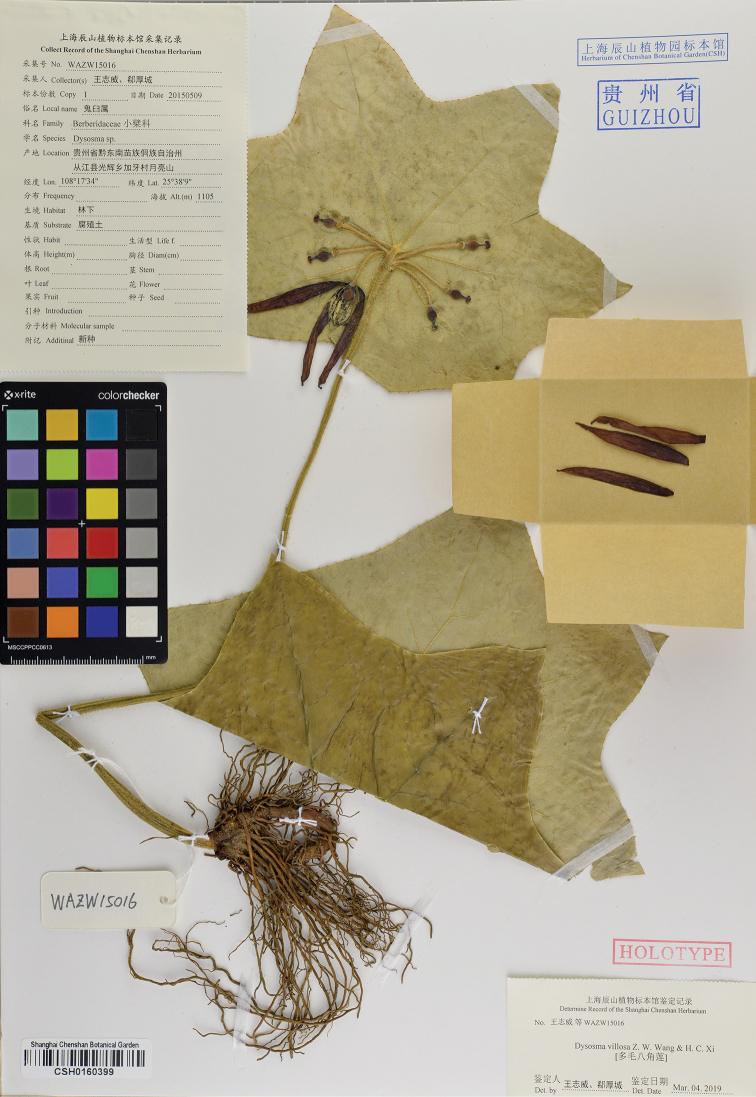
Holotype of *Dysosmavillosa* Z.W.Wang & H.C.Xi.

**Figure 2.**
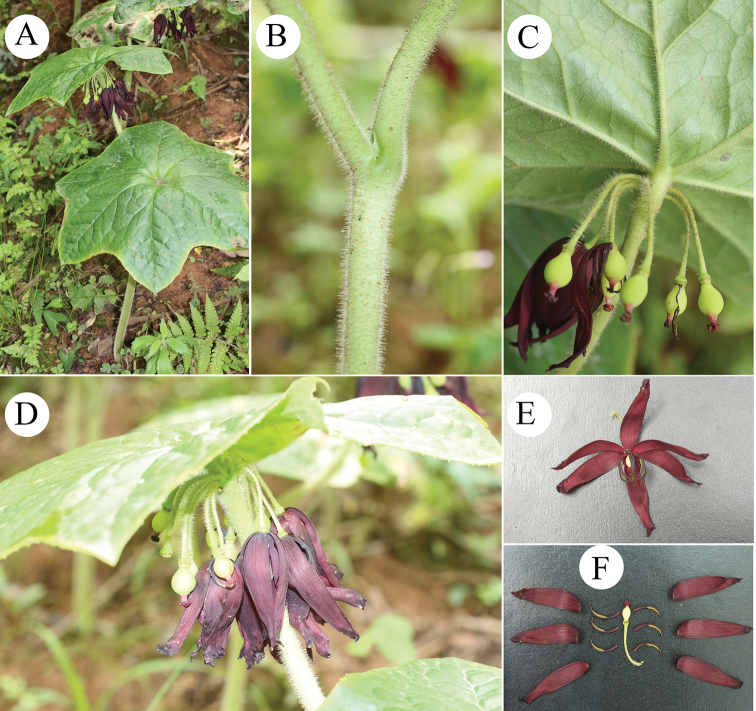
Images of living plants of *Dysosmavillosa* Z.W.Wang & H.C.Xi. **A** Plant **B** petiole and stem **C** abaxial leaf blade **D** inflorescence **E** flower **F** anatomy of flower.

**Figure 3.**
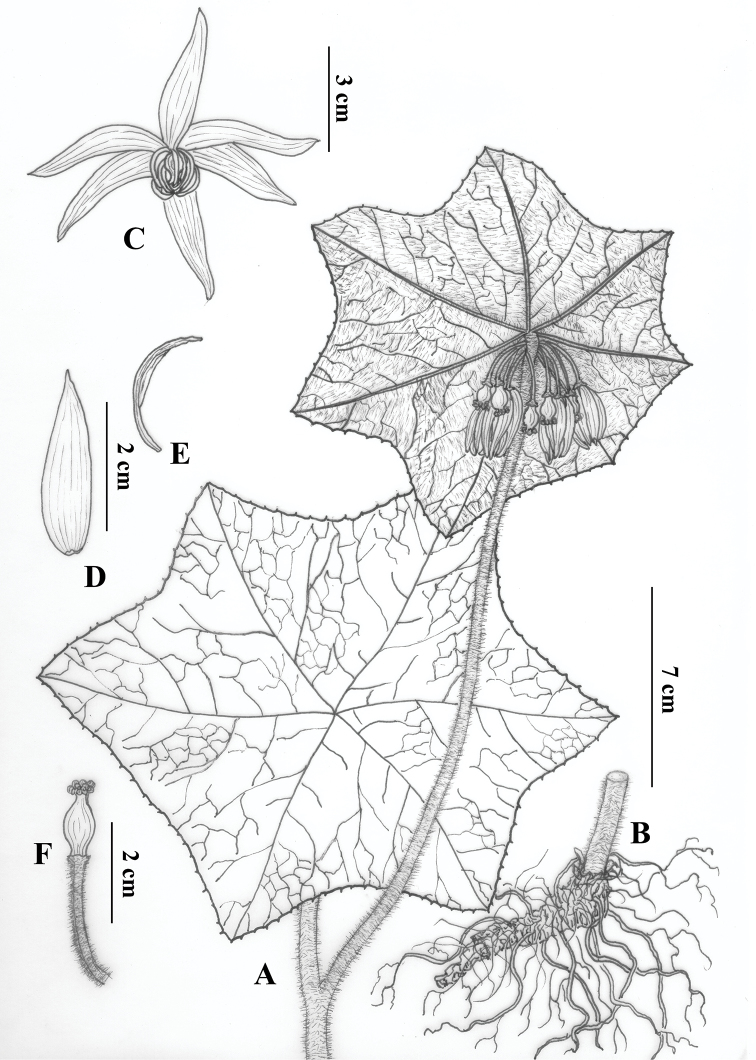
*Dysosmavillosa* Z.W.Wang & H.C.Xi. **A** Plant (aerial part) **B** root **C** flower **D** sepal **E** stamen **F** pistil.

## Discussion

*Dysosmavillosa* shares certain characteristics with *D.difformis* in having alternate leaves, leaf blades not deeply divided and/or undivided, inflorescence attached near the base of leaf blade, and oblong-loriform petals. However, it can be promptly recognised by its inflorescences generally having more flowers, and densely white-villose stems, petioles and abaxial leaf blade. Particularly, its densely white-villose stems, petioles and abaxial leaf blade and dark purple-red stigma are significantly different from *D.difformis*, as well as other species of *Dysosma*. In addition, it is worth mentioning that there are still three uncertain species described under *Podophyllum* (*P.glaucescens*, *P.hemsleyi*, and *P.trilobulum*) that probably belong to *Dysosma* (Shaw 2002, Ying et al. 2011). However, they are also found to be significantly different from *D.villosa* after we checked their morphological description (Ying et al. 2011) and images of type specimens from JSTOR Global Plants (http://plants.jstor.org). For instance, though the stem and petioles of *P.trilobulum* were also found with hairs, it was significantly different from *D.villosa* due to its trilobulate lobed leaves, inflorescence with fewer flowers (2–5) and the position of inflorescence (inserted at or above the midpoint on the petiole of the upper leaf). Comparisons of the key characters amongst *D.villosa*, *D.difformis* and the three insufficiently recognised species are listed in Table 1.

**Table 1.** Morphological comparison of key characters amongst *Dysosmavillosa*, *D.difformis* and the three insufficiently known species of *Podophyllum*.

Characters	* D. villosa *	* D. difformis *	* P. glaucescens *	* P. hemsleyi *	* P. trilobulum *
Plant height	9–23 cm tall	15–30 cm tall	up to 40 cm tall	up to 40 cm tall	20–25(–40) cm tall
Leaf blade	abaxially densely white-villous, adaxially glabrous; not deeply divided or undivided	glabrous; not deeply divided or undivided	glabrous; lower leaf 4-lobed; upper leaf with 4 obvious and 2 obscure lobes	glabrous; lobes spatulate-oblong, lobed to 4/5 of radius	glabrous; lobes trilobulate, lower leaf lobes 7, upper leaf lobes 5
Stem and Petiole	densely white-villous	glabrous	glabrous	glabrous	with fine short hairs
Inflorescence	attached near base of blade, 5–13-flowered	attached near base of blade, 2–5-flowered	inserted on petiole of upper leaf ca. 2 cm below blade, 7-flowered	inserted on petiole ca. 2 cm below blade, 4-flowered	inserted at or above midpoint on petiole of upper leaf, 2–5-flowered
Pedicel	1.6–2.2 cm long, sparsely white-villous	1–2 cm long, sparsely white-pubescent	4–6 cm long, densely hairy	3–4 cm long, glabrous	2.2–2.5 cm long, brown pilose
Petal	oblong-loriform, 4–5 × 1.2–1.6 cm	oblong-loriform, 4–5 × 0.8–1 cm	ovate-lanceolate, 10–12 × 5–6 mm	oblong-spatulate, 3–3.5 cm× 5–8 mm	ovate-lanceolate, 4–5 × 8–10 mm
Pistil	ovary obpyriform, ca. 1.2 cm long, style ca. 2 mm long, stigma crown-shaped, dark purple-red	ovary obpyriform, ca. 0.9 cm long, style ca. 2 mm long, stigma crown-shaped, yellowish	ovary ovoid, ca. 5 mm long, style 2–3 mm long, stigma peltate, corrugated, coloration unknown	ovary globose to pyriform, 6–7 mm long, style 2–3 mm long, stigma globose, corrugated, coloration unknown	unknown

### Key to the species of *Dysosma*

**Table:** 

1	Leaves opposite, inflorescence emerging at the petiole base	**2**
–	Leaves alternate, inflorescences emerging at or near the apex of the petiole	**4**
2	Lobes of leaf apically trifid; petals oblong, up to 6 cm long	*** D. delavayi ***
–	Lobes of leaf apically undivided; petals obovate-elliptic, ca. 3 cm long	**3**
3	Leaf blade glabrous, palmately-lobed, lobes triangular-ovate	*** D. pleiantha ***
–	Leaf blade pubescent on both surfaces, palmately parted, lobes cuneate-oblong	*** D. tsayuensis ***
4	Inflorescences emerging far from the leaf blade; petals obovate, 1.4–1.6 cm long	*** D. aurantiocaulis ***
–	Inflorescences emerging near the leaf blade; petals oblong, lanceolate or obovate, 2.4–10 cm long	**5**
5	Leaf lobes apically trifid; petals elliptic-lanceolate	*** D. majoensis ***
–	Leaf lobes apically undivided; petals spatulate-obovate or oblong-loriform	**6**
6	Leaves 4–9-lobed or deeply divided; petals spatulate-obovate; berries ca. 4 cm long, ellipsoid or ovoid	*** D. versipellis ***
–	Leaves not deeply divided or undivided; petals oblong-loriform; berries 1.5–2.7 cm diam., globose	**7**
7	Stems, petioles and leaf blades glabrous; inflorescences 2–5-flowered; stigma yellow	*** D. difformis ***
–	Stems, petioles and leaf blades white-villous; inflorescences 5–13-flowered; stigma dark purple-red	*** D. villosa ***

## Supplementary Material

XML Treatment for Dysosma villosa
